# Experience of Lyme disease and preferences for precautions: a cross-sectional survey of UK patients

**DOI:** 10.1186/1471-2458-13-481

**Published:** 2013-05-16

**Authors:** Afrodita Marcu, Julie Barnett, David Uzzell, Konstantina Vasileiou, Susan O’Connell

**Affiliations:** 1 Department of Information Systems and Computing, Brunel University, Kingston Lane, Uxbridge UB8 3PH, UK; 2 School of Psychology, University of Surrey, Guildford GU2 7XH, UK; 3 Formerly at the Lyme Borreliosis Unit, Health Protection Agency Microbiology Laboratory, Southampton University Hospitals NHS Trust, Southampton SO16 6YD, UK

**Keywords:** Lyme disease, Prevention, Behaviour, Precautionary measures, Ticks

## Abstract

**Background:**

Lyme disease (LD) is a tick-borne zoonosis currently affecting approximately 1000 people annually in the UK (confirmed through serological diagnosis) although it is estimated that the real figures may be as high as 3000 cases. It is important to know what factors may predict correct appraisal of LD symptoms and how the experience of LD might predict preferences for future precautionary actions.

**Methods:**

A cross-sectional survey was conducted with early LD patients via the Lyme Borreliosis Unit at the Health Protection Agency. One hundred and thirty participants completed measures of awareness of having been bitten by ticks, knowledge of ticks and LD, interpretation of LD symptoms, suspicions of having LD prior to seeing the General Practitioner (GP), and preferences for precautionary actions during future countryside visits. Chi-square tests and logistic regression were used to identify key predictors of awareness of having been bitten by ticks and of having LD. *t*-tests assessed differences between groups of participants on suspicions of having LD and preferences for future precautions. Pearson correlations examined relationships between measures of preferences for precautions and frequency of countryside use, knowledge of ticks and LD, and intentions to avoid the countryside in the future.

**Results:**

73.8% of participants (*n* = 96) reported a skin rash as the reason for seeking medical help, and 44.1% (*n* = 64) suspected they had LD before seeing the GP. Participants reporting a direct event in realizing they had been bitten by ticks (seeing a tick on skin or seeing a skin rash and linking it to tick bites) were more likely to suspect they had LD before seeing the doctor. Participants distinguished between taking precautions against tick bites *during* vs. *after* countryside visits, largely preferring the latter. Also, the more frequently participants visited the countryside, the less likely they were to endorse during-visit precautions.

**Conclusions:**

The results suggest that the risk of LD is set in the context of the restorative benefits of countryside practices, and that it may be counterproductive to overemphasize pre- or during-visit precautions. Simultaneously, having experienced LD is not associated with any withdrawal from countryside.

## Background

Reported incidence of Lyme disease (LD; also known as Lyme borreliosis) has increased in both North America and Europe, including the United Kingdom (UK). In 2011 there were 959 serologically confirmed cases in England and Wales [1], and 229 in Scotland [2] (incidence rates of 1.73 and 4.36, respectively, per 100,000 total population). Overall, the LD incidence rate in the UK is lower than that in the United States (US) of 7.8, where a total of 24,364 cases were reported in 2011 [3]. Nonetheless, LD is becoming an important issue in public health management in the UK as increasing numbers of people use the countryside for recreation.

LD, caused by pathogenic genospecies of *Borrelia burgdorferi sensu lato*, is transmitted by ticks of the *Ixodes ricinus* complex. The most common clinical feature of early LD is an erythematous skin lesion, erythema migrans (EM), with other early features including flu-like symptoms, tiredness, headaches, myalgia, and arthralgia. LD is diagnosed primarily on clinical findings and a history of tick exposure, with serological testing only being required to confirm diagnosis in patients with less specific manifestations [4].

Given the wide distribution of tick habitats [5] and frequent use of the countryside for work and recreation, current management of LD risk in the UK is primarily channelled through the provision of precautionary information to countryside visitors, typically via leaflets (see [6]). Recommended precautions usually focus on prevention of tick bites (e.g. wearing protective clothing), frequent skin checks and early removal of attached ticks. It is unclear however to what extent such risk communication practices resonate with public preferences for precautionary measures. The adoption of precautionary measures against LD has been shown to be generally low in the UK [7,8] and in other countries such as the US [9-11]. In a recent study, UK countryside visitors have been shown to be reluctant to adopt precautionary measures which they viewed as interfering with their enjoyment of nature, e.g. covering up on a warm day [12].

Nonetheless, it is important for the public to recognize accurately the symptoms of LD and seek prompt medical help, as LD can become more severe if not treated timely. Therefore, in this study we explored lay experience of contracting and being diagnosed with acute LD in order to gain insights into how precautionary information on LD might be best formulated so that the public can accurately and promptly interpret its symptoms, and what preventive measures might be seen as most preferable. We focused on what leads people to appraise their symptoms correctly by the time they consult the General Practitioner (GP) and what factors might lead to this. Given the often extended timeline between being present in a tick habitat and having a set of symptoms that prompt a visit to the GP, it is valuable to understand the optimal points for taking precautionary and preventive actions [13] as well as identifying key drivers of correct symptom appraisal.

## Methods

### Study design and setting

A cross-sectional survey was conducted in 2009 with patients aged over 16 years who had clinical manifestations consistent with early LD and positive blood tests for antibodies to *Borrelia burgdorferi*. These tests were conducted at the Lyme Borreliosis Unit at the Health Protection Agency, UK (HPA). The diagnosed patients were invited to participate in a questionnaire survey by their GP when their positive tests for LD were returned. Participants returned completed questionnaires in pre-paid envelopes. The study received ethical approval from the National Health Service (NHS) Surrey Research Ethics Committee in May 2008.

### Questionnaire measures

The survey included questions about demographics, frequency of countryside use, knowledge of ticks and LD prior to infection, open questions about symptoms that made the respondents see their GP, preferences for precautions during future visits to the countryside, and intentions to avoid the countryside in the future. The participants also indicated if they had suspected they had LD before seeing their GP: they were categorized as ‘suspecters’ if they ticked the options *Yes I was pretty sure I had Lyme disease* or *I thought it was possible I had Lyme disease*, and as ‘non-suspecters’ if they ticked the response *I had no idea at all that it could be Lyme disease*. Frequency of countryside use was assessed through the item *Approximately how often do you go to the countryside?*, which was measured on a 6-point scale ranging from 1 = *less than once a year* to 6 = *daily*.

Four items taken from the EUCALB (*European Concerted Action on Lyme Borreliosis*) environmental awareness standardised questionnaire [14] gauged participants’ assessment of what their knowledge of ticks was before they contracted LD. The items (what ticks eat, how long they feed for, whether they feed on humans, and how they get onto humans) were coded as 1 for a correct answer and 0 if incorrect, thus scores ranged from 0 to 4. Two additional items measured the participants’ estimates of pre-infection knowledge of LD (i.e. how much they thought they knew about LD and about precautions against tick bites). The answers to these two items ranged from 0 = *nothing* to 3 = *a great deal*, with scores thus ranging from 0 to 6. The six items were aggregated, creating a variable representing *Knowledge of ticks and LD* (Cronbach’s α = .64), with overall scores ranging from 0 to 10 (*Mean* = 4.66, *SD* = 2.51).

Five items representing the precautionary advice given by NHS and HPA (measured from 1 = *strongly disagree* to 5 = *strongly agree*) explored the participants’ preferences for precautions during future visits to the countryside. We did not seek to assess the applicability of these actions (e.g. whether participants did or did not walk in the middle of the path, or whether they did or did not have a dog to check for ticks). These items loaded on two factors interpreted as representing *Precautions during-visit* to the countryside and *Precautions post-visit*, respectively, see Table 1 below. A further item measured on the same 5-point scale assessed the participants’ intentions to avoid the countryside in the future, *I intend to avoid future visits to the countryside* (*Mean* = 1.13, *SD* = 0.53).

**Table 1:** Preferences for precautionary measures during future visits to the countryside following the factor analysis

**Factor**	**Items loading on each factor**	**Reliability of the factor**	**Mean (SD)**
*Precautions during-visit to the countryside*	Keep to the main paths *M* = 2.62, *SD* = 1.38, *n* = 113	*α* = . 73	*M* = 3.26, *SD* = 1.14
	Cover exposed skin with clothing *M* = 3.76, *SD* = 1.29, *n* = 124		
	Use insect repellent *M* = 3.15, *SD* = 1.35, *n* = 111		
*Precautions after-visit to the countryside*	Check dogs for ticks *M* = 3.25, *SD* = 1.37, *n* = 95	*r* = . 43	*M* = 4.04, *SD* = 0.98
	Check exposed skin for ticks *M* = 4.45, *SD* = 0.88, *n* = 128		

The respondents also indicated which of seven suggested events (*when I saw a tick on my skin*, *when I got a rash*, *when I got flu-like symptoms*, *when I looked up information about it*, *when I talked to others about it*, *when I talked to my doctor*, and *‘other reason’*) had made them realise they had been bitten by a tick. They also ranked the relative importance of the selected events from 1 = *unimportant* to 7 = *most important*. The questionnaire is provided as an Additional file 1 for the interested reader (please note that the results of some sections of the questionnaire are not reported in this paper).

### Statistical analysis

Statistical analyses were performed using the SPSS software package (SPSS Inc. Released 2009, PASW Statistics for Windows, Version 18.0, Chicago, IL, USA). The significance level was set at *p* < 0.05 on two-tailed testing. Chi-square tests were used to identify key predictors of awareness of having been bitten by ticks. We also ran a direct logistic regression to explore what predicted whether or not the participants would suspect they had LD before seeing their GP. The categorical predictor was *Direct event leading to the realization of tick bite* (1 = direct event, 0 = indirect event). The continuous predictors were *Frequency of countryside use*, *Knowledge of ticks and LD*, and *Level of education*. The categorical outcome variable was *Suspecting LD before seeing the GP* (1 = suspecter, 0 = non-suspecter). The adequacy of the expected frequencies for all pairs of categorical variables was evaluated and it was found that there were sufficient events per variable (all expected frequencies were above 1 and no more that 20% of cells had expected frequencies less than 5). The interaction terms of the *Knowledge of ticks and LD* and of the *Level of education* with their respective natural logarithms were not significant indicating that these predictors satisfied the assumption of linearity of the logit. By contrast, the interaction term of the *Frequency of countryside use* with its natural logarithm was significant (*p* = .005). Therefore, we transformed the variable by reflecting it and taking the square root, due to its moderate negative skewness, and we entered the transformed variable into the regression model. Finally, the variance inflation factors (VIF) and the tolerance statistics were obtained for the three continuous predictors to check for potential collinearity. None of these values indicated a problem of collinearity (all tolerance values > 0.2 and VIFs < 10).

Furthermore, *t*-tests were used to assess differences between groups of participants on suspicions of having LD and on preferences for future precautions. An exploratory factor analysis with Principal Component Analysis was performed on the five items pertaining to precautionary measures. Finally, Pearson product–moment correlations examined relationships between the two measures of preferences for precautions and frequency of countryside use, knowledge of ticks and LD, and intentions to avoid the countryside in the future.

## Results

### Sample description

The Lyme Borreliosis Unit distributed 333 questionnaires, of which 145 participants, 77 females and 68 males, age range years = 20 to 85, *Mean* age = 54, *SD* = 13.59, *Mdn* age = 55, responded to the survey (response rate: 43.5% of the targeted sample). The majority of participants (87.6%) were of ‘white-UK’ ethnic origin. Over three-quarters of the respondents reported visiting the countryside at least once a month (83.4% *n* = 121). Among the 188 non-responders, 40% were females (*n* = 76) and 60% males (*n* = 112). Their age range was 18 to 81 years, *Mean* = 47.41, *SD* = 16.16, *Mdn* = 47, which was significantly lower than the respondents’, *t*(328) = 6.53, *p* < .001.

### Experiencing symptoms of LD and linking them to tick bites

The majority of participants (55.9%, *n* = 81) did not suspect they had LD before seeing their GP. Among these, there were 15 participants who, at the time of completing the questionnaire, did not know that the cause of their disease was having been bitten by a tick. These participants are excluded from the analyses reported below (*n* = 130). On the basis of suspecting LD or not before seeing the doctor we categorised participants as either ‘suspecters’ (*n* = 64) or as ‘non-suspecters’ (*n* = 66) and explored whether there were differences between these two groups in terms of other measures such as reported symptoms, knowledge of ticks and LD, and preferences for future precautions.

Overall, 73.8% of participants (*n* = 96) reported the presence of an erythema migrans rash as the reason for going to the doctor. This compares to other studies which have indicated rates of EM varying from 41% of the cases [15], to 65% [16], to between 80 to 90% [17,18]. Suspecters and non-suspecters mentioned the rash in relatively equal measure, 73.4% (*n* = 47) vs. 74.2% (*n* = 49) respectively. There was no significant association between suspecting LD and reporting the presence of a rash as a reason for self-referral, so we investigated further what factors might predict the participants’ suspicions of LD before visiting the GP.

We first explored which events had been rated as the most important trigger in making the participants realize that they had been bitten by ticks (see Table 2 below).

**Table 2:** The most important events that triggered the realization of having been bitten by ticks

**Events**	**Rated as most important by suspecters (*n*** **= 64)**	**Rated as most important by non-suspecters (*n*** **= 66)**	**Total**	**Differences between suspecters and non-suspecters (Chi-square tests)**
**Direct events**	48	24	72	
*When I saw a tick on my skin*	26 (40.6%)	9 (13.6%)	35	*χ*^2^ (1) = 12.03, *p* = .001
*When I got a rash*	22 (34.4%)	15 (22.7%)	37	*χ*^2^ (1) = 2.17, ns.
*When I got flu-like symptoms*	0	0	0	N/A
**Indirect events**	16	42	58	
*When I looked up information about it*	2 (3.1%)	3 (4.5%)	5	*χ*^2^ (1) = 0.18, ns.
*When I talked to others about it*	4 (6.3%)	0	4	*χ*^2^ (1) = 4.26, *p* < .05
*When I talked to my doctor*	3 (4.6%)	31(46.9%)	34	*χ*^2^ (1) = 30.08, *p* < .001
*Other reason*	7 (10.9%)	8 (12.1%)	15	*χ*^2^ (1) = 0.05, ns.

**Figure 1:**
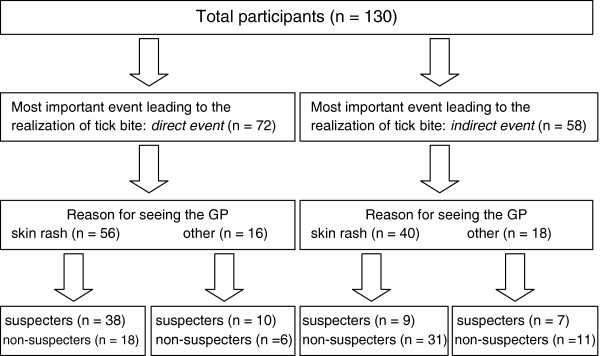
Experiencing and interpreting the symptoms of LD*. *Excludes 15 participants who did not know, at the time of the survey, that they had been bitten by ticks.

We classified the events leading to the realization that an individual had been bitten by a tick into *direct events* (*n* = 72): seeing a tick on skin, noticing a rash, getting flu-like symptoms, and *indirect events* (*n* = 58): looking up information about it, talking to others about it, talking to one’s doctor, and ‘other’. We found a significant association between the type of event leading to the realization that they had been bitten by a tick (*direct* vs. *indirect*) and suspecting LD before seeing the GP, χ^2^ (1) = 19.63, *p* < .001, *Cramer’s V* = .39, *OR* = 5.26. This means that the participants were 5.26 more likely to suspect they had LD prior to seeing their doctor if they had realized they had been bitten by a tick through a *direct event* (i.e. seeing tick attached to skin or seeing the skin rash). Figure 1 below illustrates the pathway to interpreting the symptoms of LD:

### Predicting suspicions of having LD before formal diagnosis

The results of the logistic regression showed that the full model was significantly better at predicting the probability of being a suspecter or not than the constant-only model, (*χ*^2^(4) = 36.511, *p* < .001), indicating that the predictors, as a set, reliably distinguished between suspecters and non-suspecters. Classification of case was improved, with 71.4% of suspecters and 71% of non-suspecters correctly predicted (average correct prediction: 71.2%). In addition, the Hosmer & Lemeshow test was non-significant (*χ*^2^(8) = 3.042, *p* > .05), showing that the model did not significantly differ from the observed data and fitted well. The results indicated that *Direct event* and *Knowledge of ticks and LD* were significant predictors of the likelihood of participants suspecting they had LD before seeing the GP (see Table 3).

**Table 3:** Odd ratios of suspecting LD before receiving medical help (1 = suspecter, 0 = non-suspecter)

	***B *(*SE*)**	**Odds ratio (or Exp *b*) [95% CI]**
Constant	−3.52* (1.61)	-
*Knowledge of ticks and LD*	0.21*(0.09)	1.24 [1.02; 1.50]
*Direct event leading to the realization of tick bite*	1.38**(0.42)	3.98 [1.74; 9.12]
*Age when leaving full-time education*	0.12 (0.06)	1.13 [0.99; 1.30]
*Frequency of countryside use*	−0.50(0.59)	0.60 [0.18; 1.93]

Note: *R*_*L*_^2^ = .21(Hosmer & Lemeshow), .25 (Cox & Snell), Model *χ*^2^(4) = 36.51, *p* < .001.

B: regression coefficients; SE: standard errors of regression coefficients; CI: confidence interval of odds ratio.

*p < .05; **p < .001.

The low value of *R*_*L*_^2^ (.21) suggests that only 21% of the variance in suspecting LD before visiting the GP can be accounted for by the current model. The model shows that rating a direct event (i.e., seeing a tick on skin or noticing a skin rash) as the most important trigger in realizing one has been bitten by a tick significantly predicts whether one will suspect they had LD before being formally diagnosed, over and above one’s knowledge of ticks and of LD. As the value of *exp b* indicates, the odds of suspecting one has LD were almost 4 times higher for those participants who rated a direct event as the most important trigger in realizing the tick bite than for those who did not.

### Preferences for precautionary measures in the future

The majority of participants, 91% (*n* = 119), strongly disagreed with the idea of avoiding the countryside in the future. Regarding the preferences for precautionary measures, an exploratory factor analysis indicated the presence of two factors which explained 70% of the variance (48.94% and 21.19%, respectively), and represented precautions to take *during* vs. *after* the visit to the countryside. These results suggest that participants distinguished between precautions that can be taken at different times during their activities in the countryside. Precautions during-visit to the countryside and Precautions after-visit to the countryside were positively and significantly correlated, *r* =. 49, *p* < .001. There were no significant differences between suspecters and non-suspecters regarding preferences either for precautions *during*-visit *Ms* = 3.24 vs. 3.29, *t* (123) = .25, or for precautions *after*-visit *Ms* = 4.10 vs. 3.98, *t* (126) = .49, all *p* > .05. However, regardless of their suspecter profile, the participants preferred the precautions *after*-visit to those *during*-visit, *M* = 4.02, *SE* = 0.08, vs. *M* = 3.25, *SE* = 0.10, *t*(123) = 7.93, *p* < .001, *r* = .58. Furthermore, those who visited the countryside more frequently were less likely to prefer precautionary action *during* the visit (see Table 4).

**Table 4:** Pearson correlations between the preferences for precautions against tick bites and the other dependent variables

	***Preferences for precautions in the future***	
	***During visit***	***After visit***
*Frequency of countryside use*	*r* = −.23*, *n* = 124	*r* = .06, *n* = 127
*Knowledge of ticks and LD*	*r* = −.09, *n* = 124	*r* = .09, *n* = 127
*Intentions to avoid the countryside in the future*	*r* = .22*, *n* = 123	*r* = .07, *n* = 125

*p < .05.

## Discussion

The objectives of this study were to understand the lay experience of contracting and being diagnosed with early LD, to identify key drivers of correct symptom appraisal before receiving medical help, and to explore what preventive measures might be seen as most preferable. The study found that EM, the most common clinical sign of LD, played an important role in prompting participants to seek medical help. A ‘skin rash’ was the most frequently mentioned reason for seeing the primary healthcare practitioner, reported by 73.8% of the sample. However, EM alone did not lead participants to suspect they had LD. Those participants who rated a direct event as being the most important trigger for realizing they had been bitten by a tick (seeing a tick on skin or seeing a rash) were most able to interpret their symptoms correctly before seeing their GP. In this study, 49.2% of the sample suspected they had LD prior to receiving medical help, mainly because they had seen a tick attached to their skin. The suspecters more often rated the direct events leading to the realization of being bitten by ticks as being important compared to non-suspecters – the latter only realising they had been bitten by ticks after talking to their GP. While suspecters and non-suspecters reported in relatively equal measure the presence of a skin rash as the reason to see their GP, only those who knew they had been bitten by ticks were able to correctly interpret their symptoms before being formally diagnosed. We would argue that suspecting LD before receiving medical help is important because it can assist with patient-doctor communication and help GPs acquire the relevant history of patients’ exposure to tick bites that is necessary for the diagnosis of LD [13]. As for the role of factual knowledge of ticks and LD, this was also found to have contributed to suspicions of having LD before seeing the GP, indicating that the suspecters may have known more about ticks and LD than the non-suspecters before they acquired LD. However, it is perhaps difficult and somewhat speculative to ascertain the participants’ knowledge of ticks and LD prior to becoming infected, as the boundaries between previous and subsequent knowledge can become blurred in retrospect. Given this limitation in assessing previous knowledge, it is perhaps more important to establish the events that led the participants to realise they had been bitten by ticks. Our results showed that the experience of a direct event in the realization of tick bites predicted suspicions of having LD over and above one’s knowledge of ticks and LD. Apart from being more robust than assessing knowledge, this measure suggests that knowledge may not be enough in interpreting one’s symptoms and at the same time underlines the importance of checking one’s skin for tick bites.

Regarding the adoption of precautionary measures during future visits to the countryside, the present survey revealed a number of significant findings. Firstly, participants distinguished between *during*-visit and *after*-visit precautions, primarily preferring the latter. This finding ties in with other studies which have shown that checking oneself for ticks after being in tick-populated areas was the most frequently reported precautionary behaviour against LD [19]. Secondly, the more the participants used the countryside, the less likely they were to endorse the notion of during-visit precautions. As the majority of participants were frequent countryside users (83.4% visited the countryside at least twice a month), the findings would suggest that it is the population most at risk of tick bites and LD that may be less likely to adopt during-visit precautions. Coupled with the findings that the *after*-visit precautions were preferred to the *during-*visit ones, it could be argued that effective risk communication for the population at risk of LD should emphasize *after*-visit precautions, primarily checking oneself for tick bites after being in the countryside. Thirdly, as the present findings indicated, noticing a tick on one’s skin or noticing a skin rash and deducing that one has been bitten by ticks enabled the participants to interpret their symptoms correctly and to suspect they had LD before diagnosis. Arguably, the perceived effectiveness of after-visit precautions may have underpinned the respondents’ higher preference for them.

The strengths of this study lie in the fact that it was conducted with a sample that had been diagnosed with early LD following serological testing. Our recruitment of participants supplemented that of other studies where only clinical criteria were used in the diagnosis of EM. Empirical studies on medically *bona fide* diagnoses of LD are important as they provide an important balance to studies focusing on the ‘chronic’ nature, treatment, or long-term effects of LD [20,21]. On the negative side, this study was cross-sectional and therefore we could not explore how the experience of LD subsequently influenced the participants’ actual adoption of precautions and use of the countryside.

## Conclusions

Knowledge and adoption of precautions against LD are generally low among the lay public, and current management of the risk of LD involves information provision aimed at behaviour change. To our knowledge, this is the first study that indicates a distinction between lay preferences for LD precautions *during* vs. *after* visit to the countryside, with the higher preferences for the latter indicating that precautions are understood within the restorative context of the countryside. The present study suggests if LD risk communication practices are to be attuned to public preferences for precautions, risk communicators should not neglect to emphasise the value of the precautions that can be taken after visiting the countryside.

## Abbreviations

LD: Lyme disease; EM: Erythema migrans; GP: General practitioner; HPA: Health Protection Agency; NHS: National Health Service; UK: United Kingdom; US: United States; EUCALB: European Concerted Action on Lyme Borreliosis.

## Competing interests

The authors declare that they have no competing interests.

## Authors’ contributions

AM contributed to the design of the study, collected the data, performed the analysis and interpretation of the data, and drafted the manuscript. JB contributed to the design of the study, the interpretation of the data, and the preparation of the manuscript. DU contributed to the design of the study, the interpretation of the data, and the preparation of the manuscript. KV contributed to the analysis and interpretation of the data, and the preparation of the manuscript. SO’C contributed to the design of the study, acquired the data, provided clinical expertise regarding LD, and revised the manuscript. All authors read and approved the final manuscript.

## Pre-publication history

The pre-publication history for this paper can be accessed here:

http://www.biomedcentral.com/1471-2458/13/481/prepub

## Supplementary Material

Additional file 1 Study questionnaire. Click here for file
