# Supplementary material for: Experience of Lyme disease and preferences for precautions: a cross-sectional survey of UK patients
Source: BMC Public Health. 2013 May 16;13:481. doi: 10.1186/1471-2458-13-481 (PMC3681680; doi:10.1186/1471-2458-13-481)
Supplement: Additional file 1 — Study questionnaire. [file 1471-2458-13-481-S1.doc]

*Assessing and communicating animal disease risks*

*for countryside users*

Dear Participant,

Thank you for agreeing to take part in our research. As you know from the information sheet, we have invited you to take part because your doctor has diagnosed you as testing positive for *Borrelia burgdorferi* antibodies, the clinical sign of Lyme disease.

We believe that it will take you about 15-20 minutes to complete the questionnaire. Please start at the beginning and work your way through it in the order in which the questions are presented. Please see the other side of this page for guidelines on how to fill in the questionnaire.

We would like you to answer all relevant questions but of course if there are any you would rather not answer you can simply leave them blank.

THANK YOU!

THE RESEARCH TEAM, UNIVERSITY OF SURREY

**HOW TO FILL IN THIS SURVEY QUESTIONNAIRE**

**1.** Most of the items have one or more boxes for your answer. The questionnaire will be scanned by a computer – so mark the boxes of your choice with **one diagonal line** as shown in the examples below. Please use **black ink or biro**.

Example:

**2.** If you make a mistake or wish to change your answer, please fill in **all** of the box containing the error, and then put a diagonal line to correspond with your new answer.

Example:

1. Some items will ask you to indicate your level of agreement by marking the box in the column which best reflects your answer. The columns are titled, for example, “strongly agree” to “strongly disagree”. In the following example, the answers would be “Strongly agree” for the first item and “Somewhat disagree” for the second item.

Example: Strongly Somewhat Neutral Somewhat Strongly

disagree disagree agree agree

## THANK YOU VERY MUCH FOR YOUR HELP

**A. ABOUT YOU**

*First of all, please answer the following questions about yourself:*

| **1. What is your gender?** | Female | ⬜ | Male | ⬜ |
| --- | --- | --- | --- | --- |
| **2. What is your age in years?** |  | | |  |

**3. What is the first half of your postcode?**

**4. At what age did you finish full time education, or are you still in full time education?**

| Still in full time education | Don't  Know |
| --- | --- |
| ⬜ | ⬜ |

| **5. In which ethnic category would you class yourself?** | | | | | | | | | | | | |  |
| --- | --- | --- | --- | --- | --- | --- | --- | --- | --- | --- | --- | --- | --- |
| White (UK origin) | ⬜ | | White (other) | | ⬜ | Chinese | | ⬜ | | Indian | | ⬜ |  |
| Black (Caribbean) | ⬜ | | Black (other) | | ⬜ | Bangladeshi | | ⬜ | | Pakistani | | ⬜ |  |
| Black (African) | ⬜ | | Other | |  |  | |  | |  | |  |  |
| **6. Approximately how often do you go to the countryside?** (When we say countryside in this questionnaire we mean forests, woods, fields, parks, heath or moorland) | | | | | | | | | | | | | |
| Daily | | Once or twice a week | | Once or twice a month | | | Once or twice every few months | | Once or twice a year | | Less than once a year | | |
| ⬜ | | ⬜ | | ⬜ | | | ⬜ | | ⬜ | | ⬜ | | |

1. **YOUR EXPERIENCES – LOOKING BACK**

We know that people with Lyme disease often do not realise that they have been bitten by a tick or know exactly when this happened.

| **7. At the time of filling in this questionnaire, did you know that the likely cause of your symptoms was having been bitten by a tick?** | Yes | No |
| --- | --- | --- |
|  | ⬜ | ⬜ |

*If you answered No, please go to Question 22; If you answered Yes please go to Question 8*

**8.** **In the column on the left, please mark all the events that made you realise that you had been bitten by a tick. In the right hand column, please indicate how important each event you marked in the first column was in making you realise you had been bitten by a tick*.*** (1 = most important, 2 = next most important etc).

| **Events that made you realise you had been bitten** |  | **Order of importance**  *(1=most important*  *2= next most important etc.)* | | |
| --- | --- | --- | --- | --- |
| ⬜ | When I saw a tick on my skin................................ | ⬜ | | |
| ⬜ | When someone else saw a tick on my skin.......... | ⬜ | | |
| ⬜ | When I got a rash................................................. | ⬜ | | |
| ⬜ | When I got flu like symptoms................................. | ⬜ | | |
| ⬜ | When I looked up information about it................... | ⬜ | | |
| ⬜ | When I talked to others about it............................. | ⬜ | | |
| ⬜ | When I talked to my doctor..................................... | ⬜ | | |
| ⬜ | Other reason........................................................... | ⬜ | | |
| ********If you marked ‘other reason’ please briefly explain what this was.* | | | |  |
|  | | | |  |
| **9. Looking back, where do you think you were when you were bitten by a tick?**  *Please write the name of the place (for example, Alice Holt Forest, Hampshire). If you have no idea at all, just leave it blank.* | | |  | |
|  | | |  | |

| **10. Around the time you were bitten by a tick, had you heard of Lyme disease?** |  | Yes | No | Can’t remember |
| --- | --- | --- | --- | --- |
|  |  | ⬜ | ⬜ | ⬜ |

| **11. How much do you think that you knew about Lyme disease at the time you were bitten?** | Nothing | Not a lot | Quite a lot | A great deal |
| --- | --- | --- | --- | --- |
|  | ⬜ | ⬜ | ⬜ | ⬜ |

| **12. Around the time of the tick bite, did you know anything about precautions that can be taken to decrease the likelihood of being bitten by a tick?** |  | Yes | No | Can’t remember |
| --- | --- | --- | --- | --- |
|  |  | ⬜ | ⬜ | ⬜ |

| **13. If yes, how much do you think that you knew about these precautions at that time?** | Nothing | Not a lot | Quite a lot | A great deal |
| --- | --- | --- | --- | --- |
|  | ⬜ | ⬜ | ⬜ | ⬜ |

Keep thinking back to around the time you were bitten by a tick. *Please answer the following questions.*

| **14. Around the time of the tick bite, had you ever heard of a creature called a tick?** |  | Yes | No | Can’t remember |
| --- | --- | --- | --- | --- |
| *If No, go to Q21* |  | ⬜ | ⬜ | ⬜ |

| **15. What did you think ticks eat?** |  | Leaves | Sap | Blood |
| --- | --- | --- | --- | --- |
|  |  | ⬜ | ⬜ | ⬜ |
|  |  | Pollen | Other insects | Don’t know |
|  |  | ⬜ | ⬜ | ⬜ |

| **16. How long did you think ticks feed for?** | Seconds | Minutes | Days | Don’t know |
| --- | --- | --- | --- | --- |
|  | ⬜ | ⬜ | ⬜ | ⬜ |

| **17. Around the time of the tick bite, did you know ticks feed on humans?** |  | Yes | No | Can’t remember |
| --- | --- | --- | --- | --- |
| *(If No, go to Q19)* |  | ⬜ | ⬜ | ⬜ |

| **18. If ‘yes’, how did you think ticks get on to humans?** *(mark more than one if you wish)* | Land from flight | Crawl from vegetation | Drop from trees | Don’t know |
| --- | --- | --- | --- | --- |
|  | ⬜ | ⬜ | ⬜ | ⬜ |

| **19. Around the time of the tick bite, did you know that ticks can transmit Lyme disease?** |  | Yes | No | Can’t remember |
| --- | --- | --- | --- | --- |
| *If No, go to Q21* |  | ⬜ | ⬜ | ⬜ |

| **20. If yes, how did you find out that ticks transmit Lyme disease?** (Did someone tell you? Did you read it somewhere?) *Please write a brief explanation* |
| --- |
|  |
|  |

**21. Please think back to the time before you might have been bitten by a tick and indicate how you much you would have agreed with each of the following statements at that time.**

|  | Strongly  disagree | Somewhat  disagree | Neutral | Somewhat  agree | Strongly  agree |
| --- | --- | --- | --- | --- | --- |
| I am worried about the risk of being bitten by a tick......................... | ⬜ | ⬜ | ⬜ | ⬜ | ⬜ |
| In the countryside there is only a small chance of being bitten by a tick.................................................................................................... | ⬜ | ⬜ | ⬜ | ⬜ | ⬜ |
| Being bitten by a tick could have serious health consequences.... | ⬜ | ⬜ | ⬜ | ⬜ | ⬜ |
| I dread being bitten by a tick............................................................ | ⬜ | ⬜ | ⬜ | ⬜ | ⬜ |
| The risks of tick bites are well known to the people that are exposed to them............................................................................. | ⬜ | ⬜ | ⬜ | ⬜ | ⬜ |
| I can control my exposure to the risk of being bitten by a tick.......... | ⬜ | ⬜ | ⬜ | ⬜ | ⬜ |

**22. Think back to the events leading to your diagnosis with the clinical signs of Lyme disease. What was it that first made you decide to go to the doctor’s?** *Please explain.*

|  |
| --- |
|  |

**23. Before you first went to the doctor’s, did you think that you might have Lyme disease?**

| Yes I was pretty sure I had Lyme disease | I thought it was possible I had Lyme disease | I had no idea at all that it could be Lyme disease |
| --- | --- | --- |
| ⬜ *Go to Q 24* | ⬜ *Go to Q 24* | ⬜ *Go to Q 25* |

**24. What made you suspect you might have Lyme disease?**

|  |
| --- |
|  |

**C. DIAGNOSIS**

**25. Please think back to the time before you were diagnosed by the doctor as positive for *Borrelia burgdorferi* antibodies - the clinical sign of Lyme disease. Indicate your agreement with each of the following statements:**

|  | Strongly  disagree | Somewhat  disagree | Neutral | Somewhat  agree | Strongly  agree |
| --- | --- | --- | --- | --- | --- |
| It was important for me to try and find out what might be causing my health condition............................................................................ | ⬜ | ⬜ | ⬜ | ⬜ | ⬜ |
| I was not satisfied with what I knew about my health condition......... | ⬜ | ⬜ | ⬜ | ⬜ | ⬜ |
| I was confident I knew the best places to look for reliable information about my health condition............................................... | ⬜ | ⬜ | ⬜ | ⬜ | ⬜ |
| I felt satisfied that I knew all I needed to about my health condition. | ⬜ | ⬜ | ⬜ | ⬜ | ⬜ |
| I did not feel I needed to find out more myself about my health condition............................................................................................ | ⬜ | ⬜ | ⬜ | ⬜ | ⬜ |
| I was worried that I did not know more about my health condition... | ⬜ | ⬜ | ⬜ | ⬜ | ⬜ |
| I knew that I had friends or family that I could ask about my health condition........................................................................................... | ⬜ | ⬜ | ⬜ | ⬜ | ⬜ |
| I was concerned about how little I knew about my health condition............................................................................................ | ⬜ | ⬜ | ⬜ | ⬜ | ⬜ |
| I was not sure how to go about finding out more about my health  condition............................................................................................. | ⬜ | ⬜ | ⬜ | ⬜ | ⬜ |

**D. LOOKING FOR INFORMATION**

**26. In the period leading up to your diagnosis as positive for *Borrelia burgdorferi* antibodies, we would like to know which places, people or sources you used to find out what may have been the matter with you (apart from your GP). In the boxes on the left, please mark which of the following information sources you used. In the boxes on the right, please indicate how *much you used* each source of information.**

| Information sources you used |  | How ***much*** you used the information source | | | |
| --- | --- | --- | --- | --- | --- |
|  |  | Not much | A little | Quite a lot | A great deal |
| ⬜ | Magazines................................................... | ⬜ | ⬜ | ⬜ | ⬜ |
| ⬜ | Official Information leaflets........................ | ⬜ | ⬜ | ⬜ | ⬜ |
| ⬜ | Newspapers................................................ | ⬜ | ⬜ | ⬜ | ⬜ |
| ⬜ | Books......................................................... | ⬜ | ⬜ | ⬜ | ⬜ |
| ⬜ | TV and radio.............................................. | ⬜ | ⬜ | ⬜ | ⬜ |
| ⬜ | Friends and family....................................... | ⬜ | ⬜ | ⬜ | ⬜ |
| ⬜ | Someone with experience of Lyme disease | ⬜ | ⬜ | ⬜ | ⬜ |
| ⬜ | NHS Direct.................................................. | ⬜ | ⬜ | ⬜ | ⬜ |
| ⬜ | The Health Protection Agency.................... | ⬜ | ⬜ | ⬜ | ⬜ |
| ⬜ | Other medical websites.......................... | ⬜ | ⬜ | ⬜ | ⬜ |
| ⬜ | Countryside staff (e.g. Visitor Centres)....... | ⬜ | ⬜ | ⬜ | ⬜ |
| ⬜ | Patient groups.............................. | ⬜ | ⬜ | ⬜ | ⬜ |
| ⬜ | Other........................................................... | ⬜ | ⬜ | ⬜ | ⬜ |

**If you marked ‘other’ please say which other information sources you have used**

|  |
| --- |
|  |

**27. Now, for each information source that you indicated in Question 26 you had used please tell us how much it helped you *improve your understanding* of what might be wrong.**

|  | How much the information source  ***helped you improve your understanding*** | | | |
| --- | --- | --- | --- | --- |
|  | Not at all | A little | Quite a lot | A great deal |
| Magazines....................................................................................... | ⬜ | ⬜ | ⬜ | ⬜ |
| Official Information leaflets............................................................. | ⬜ | ⬜ | ⬜ | ⬜ |
| Newspapers.................................................................................... | ⬜ | ⬜ | ⬜ | ⬜ |
| Books.............................................................................................. | ⬜ | ⬜ | ⬜ | ⬜ |
| TV and radio.................................................................................... | ⬜ | ⬜ | ⬜ | ⬜ |
| Friends and family........................................................................... | ⬜ | ⬜ | ⬜ | ⬜ |
| Someone with experience of Lyme disease.................................... | ⬜ | ⬜ | ⬜ | ⬜ |
| NHS Direct...................................................................................... | ⬜ | ⬜ | ⬜ | ⬜ |
| The Health Protection Agency......................................................... | ⬜ | ⬜ | ⬜ | ⬜ |
| Other medical websites................................................................... | ⬜ | ⬜ | ⬜ | ⬜ |
| Countryside staff (e.g. Visitor Centres)........................................... | ⬜ | ⬜ | ⬜ | ⬜ |
| Patient groups................................................................................. | ⬜ | ⬜ | ⬜ | ⬜ |
| Other............................................................................................... | ⬜ | ⬜ | ⬜ | ⬜ |

**28.** **Now, for each information source that you indicated in Question 26 you had used please tell us how much it helped *you identify the best actions to take*.**

|  | How much the information source  ***helped you identify the best actions to take*** | | | |
| --- | --- | --- | --- | --- |
|  | Not at all | A little | Quite a lot | A great deal |
| Magazines....................................................................................... | ⬜ | ⬜ | ⬜ | ⬜ |
| Official Information leaflets.............................................................. | ⬜ | ⬜ | ⬜ | ⬜ |
| Newspapers.................................................................................... | ⬜ | ⬜ | ⬜ | ⬜ |
| Books.............................................................................................. | ⬜ | ⬜ | ⬜ | ⬜ |
| TV and radio.................................................................................... | ⬜ | ⬜ | ⬜ | ⬜ |
| Friends and family........................................................................... | ⬜ | ⬜ | ⬜ | ⬜ |
| Someone with experience of Lyme disease.................................... | ⬜ | ⬜ | ⬜ | ⬜ |
| NHS Direct...................................................................................... | ⬜ | ⬜ | ⬜ | ⬜ |
| The Health Protection Agency........................................................ | ⬜ | ⬜ | ⬜ | ⬜ |
| Other medical websites................................................................... | ⬜ | ⬜ | ⬜ | ⬜ |
| Countryside staff (e.g. Visitor Centres)........................................... | ⬜ | ⬜ | ⬜ | ⬜ |
| Patient groups............................................................................... | ⬜ | ⬜ | ⬜ | ⬜ |
| Other............................................................................................... | ⬜ | ⬜ | ⬜ | ⬜ |

**29. Now, for each information source that you indicated in Question 26 you had used please tell us how much it helped *reassure any concerns you had*.**

|  | How much the information source  ***helped*** ***reassure any concerns you had*** | | | |
| --- | --- | --- | --- | --- |
|  | Not at all | A little | Quite a lot | A great deal |
| Magazines....................................................................................... | ⬜ | ⬜ | ⬜ | ⬜ |
| Official Information leaflets............................................................. | ⬜ | ⬜ | ⬜ | ⬜ |
| Newspapers.................................................................................... | ⬜ | ⬜ | ⬜ | ⬜ |
| Books.............................................................................................. | ⬜ | ⬜ | ⬜ | ⬜ |
| TV and radio................................................................................... | ⬜ | ⬜ | ⬜ | ⬜ |
| Friends and family........................................................................... | ⬜ | ⬜ | ⬜ | ⬜ |
| Someone with experience of Lyme disease.................................... | ⬜ | ⬜ | ⬜ | ⬜ |
| NHS Direct...................................................................................... | ⬜ | ⬜ | ⬜ | ⬜ |
| The Health Protection Agency....................................................... | ⬜ | ⬜ | ⬜ | ⬜ |
| Other medical websites........................................................ | ⬜ | ⬜ | ⬜ | ⬜ |
| Countryside staff (e.g. Visitor Centres)......................................... | ⬜ | ⬜ | ⬜ | ⬜ |
| Patient groups............................................................................... | ⬜ | ⬜ | ⬜ | ⬜ |
| Other............................................................................................... | ⬜ | ⬜ | ⬜ | ⬜ |

**30. If you indicated in the questions above that you got information from patient groups or from other medical websites please say which ones.**

|  |
| --- |
| Other medical websites:  Patient groups: |

**E. FINALLY…LOOKING AHEAD**

| **31. Please indicate how much you agree or disagree with the following statements:**  *In the next 3 months when I visit the countryside I intend to…* |
| --- |

|  | Strongly  disagree | Somewhat  disagree | Neutral | Somewhat  agree | Strongly  agree |
| --- | --- | --- | --- | --- | --- |
| Keep to the main paths.................................................................. | ⬜ | ⬜ | ⬜ | ⬜ | ⬜ |
| Cover exposed skin with clothing.................................................. | ⬜ | ⬜ | ⬜ | ⬜ | ⬜ |
| Use insect repellent....................................................................... | ⬜ | ⬜ | ⬜ | ⬜ | ⬜ |
| Check dogs for ticks....................................................................... | ⬜ | ⬜ | ⬜ | ⬜ | ⬜ |
| Check exposed skin for ticks.......................................................... | ⬜ | ⬜ | ⬜ | ⬜ | ⬜ |

**Please indicate how much you agree or disagree with the following statements:**

|  | Strongly  disagree | Somewhat  disagree | Neutral | Somewhat  agree | Strongly  agree |
| --- | --- | --- | --- | --- | --- |
| **32.** I intend to avoid future visits to the countryside.................... | ⬜ | ⬜ | ⬜ | ⬜ | ⬜ |
| **33.** In future if I saw a tick was attached to my skin I would  remove it myself………………………………………………………. | ⬜ | ⬜ | ⬜ | ⬜ | ⬜ |

| **34. Finally, was there a particular time or place when you think that there should have been more information available about ticks and how best to protect yourself from being bitten?** | |
| --- | --- |
|  |  |
| Yes, I think so.................. ⬜ | No, I don’t think so...................... ⬜ |

| **35. If you said ‘yes, I think so’, please write a few lines explaining.** | |
| --- | --- |
|  | |
| i) what you think you should have been told   |  | | --- | |  | |  |
| ii) who should have given you this information   |  | | --- | |  | |  |
| iii) where you think it should have been available |  |

|  |
| --- |
|  |

**Thank you very much for your help!**

**If there is anything else that you would like to tell us, please write on the other side of this page.**

**If you have any other comments, please write here.**

Thank you very much for completing this questionnaire.

We are conducting a further study which involves conducting some telephone interviews lasting for about 45-50 minutes.

If you are willing to be interviewed and would like further information please fill in your contact details below and return the form to us along with your questionnaire.

In the interview we would be asking you for some more details about your experience of acquiring Lyme disease, and about the sorts of information that you have found helpful. We would also like to know your views about how this information is best provided to the public more generally.

All the information that you provide will be held and processed in the strictest confidence, and in accordance with the Data Protection Act (1998). Your name will be replaced with a pseudonym in the interview transcription and analysis. We will only use your data to meet the aims and objectives of this project and will under no circumstances provide any of your details to third parties.

If you would like take part in this study please complete your details below.

How would you like us to contact you? *(Please complete whichever sections you wish)*

Postal address: 1^st^ line ___________________________________

2^nd^ line ___________________________________

Town ___________________________________

Postcode ___________________________________

Tel No: Home ___________________________________

Mobile ___________________________________

Email: ___________________________________
